# Reports of Cases Occurring in the Medical Ward of the Buffalo Hospital

**Published:** 1858-04

**Authors:** A. W. Nichols

**Affiliations:** Assistant to Chair of Clinical Medicine, University of Buffalo


④ BUFFALO MEDICAL JOURNAL

AND

MONTHLY REVIEW.

**VOL. 13.** APRIL, 1858. **NO. 11.**

### ORIGINAL COMMUNICATIONS.

ART. I.—*Abstract of Clinical Lectures delivered at the Buffalo Hospital of the Sisters of Charity, upon the Diseases treated in the Male Medical Ward, by* AUSTIN FLINT, M. D., *Attending Physician, during the present College Session, viz., from October* 14*th*, 1857, *to February* 27, 1858. Reported by A. W. NICHOLS, M. D., Assistant to Chair of Clinical Medicine, University of Buffalo.

No. V. *For February.*

*Pulmonary Tuberculosis: a New and Reliable Physical Sign of Excavation; Effect of Hæmoptysis on the Progress of the Disease; Period of Occurrence, with Cases happening late in Life; Duration of the Disease; Treatment by Sustaining Measures, with Palliatives. Acute Phthisis, Case of. Interesting Cases illustrative of the foregoing, &c.*

The subject of phthisis, or tuberculous disease of the lungs, is by no means exhausted. Within quite a recent period, more correct ideas respecting the nature of tuberculosis have become prevalent, and the symptoms and physical signs of the disease have been more carefully studied, especially in reference to the diagnosis of tuberculosis in its earliest stages. The methods of treatment, also, have been materially improved; and, in not a few instances, individuals, by more judicious management, have been restored to a comparative degree of health. Although so much attention has been paid to the pathology, diagnosis, and the treatment of phthisis, yet there remains many matters of interest and of importance to be determined by future observation and reflection.

It is not our object at present to enter into a detailed account of this disease, but simply to discuss a few points of practical and of scientific interest, relating to the physical signs, the symptoms, and the treatment of tuberculosis of the lungs; and to bring forward a few of the more striking cases that have been admitted to the ward, during the past winter, as illustrations of the subject under consideration.

Notwithstanding the numerous discoveries in physical exploration of the chest, heretofore we have not been in the possession of a reliable physical sign indicative of a cavity or cavities in the lungs. As a matter of practical importance relating either to diagnosis or to treatment, this is of comparatively little consequence; for the condition of the patient, and other physical signs, usually render the diagnosis sufficiently accurate; and as for the treatment, the general measures, (as we shall see hereafter,) applicable to every stage of the disease, are more imperatively demanded at this period. As a matter of scientific interest, however, the subject claims our attention.

If we turn back to ascertain the views of the discoverer of auscultation, we shall find that he regards pectoriloquy as the physical sign most distinctive of a cavity, and considers its presence almost positive evidence of an excavation. In treating of bronchial and cavernous respiration, Laennec insists on the importance of distinguishing the former from the vesicular respiration. But, in comparing the cavernous with the bronchial respiration, he says, "This variety has the same characters as the preceding, (bronchial.) only that it further convers the idea of air entering into a larger cavity than a bronchial tube." And to show that the distinction between the two was not always readily drawn, he adds, "and when there exists any doubt," &c.* It is evident, that Laennec regarded cavernous respiration as a variety only of the bronchial respiration, and not as an entirely different kind of respiration.

Passing from Laennec to a distinguished investigator of diseases of the chest, in more recent times, we find Walshe describing the respiratory sound proceeding from a cavity, as "hollow, hoarse, blowing, and cavernous, or

* Læennec on the Chest. Translated by Forbes. 3d revised London ed. 1830.

actually amphoric." He speaks of the difficulty of hearing a pure cavernous respiration, on account of its characters being marked by the "diffused blowing," (diffused bronchial respiration,) "which is the result of the condensation existing around the excavations." The cavernous respiration is hollow, the pitch *lower* than in tubular (or bronchial) respiration, and not as quickly produced or evolved. This kind of respiration, he remarks, is often deficient where excavations exist. But he believes that it is never present where excavations are absent.* It will be seen that some advance has been made towards determining the distinctive characters of the cavernous respiration, since the time of Laennec. But, Dr. Walshe does not make mention of any difference in the characters of the inspiratory and expiratory sounds in cavernous respiration.

Skoda, writing about the same time, states that "a cavity in a lung does not always yield the same murmur." He speaks of the various physical conditions of excavation; and endeavors to determine the characters of the various sounds by the "laws of consonance," of which theory he is the great advocate. But, the nearest approach he makes to any thing definite, is in what he says concerning certain cavities with membranous walls, where the surrounding lung is not solidified to any great extent. "In such cases the air causes a feeble murmur, not resembling the vesicular, but *something between an aspiration and a blowing.*" To sum up, he concludes, "that percussion and auscultation afford very few certain signs of the presence of a cavity."†

As long ago as 1852, Prof. Flint described the cavernous respiration as a distinct type of the respiratory sound; and clearly pointed out the characters of the inspiration and of the expiration.‡ These views are developed more at length in a more recent work.§ Since the publication of this work abundant opportunities have been afforded of verifying the statements regarding the cavernous respiration, as well as of developing more fully the characters of a kind of respiration mentioned in the above work as the *broncho-cavernous.*

Having already consumed so much space in the consideration of the opinions of the authors referred to, we refer the reader to Prof. F.'s work, for a complete description of the cavernous respiration. We shall merely recapit-

* Walshe on the Heart and Lungs. London edition of 1854.

† Skoda. Translated by Markham. Published at Philadelphia, 1854.

‡ Vide *Prize Essay,* 1852.

§ Flint on the Respiratory Organs. 1856.

ulate the distinctive qualities of the bronchial and of the cavernous respirations as described by Dr. F.

In *bronchial respiration*, the sound is tubular, and there is an interval between the sound of inspiration and that of expiration; the inspiration is high in pitch, shortened in duration, and rapidly evolved; whilst the expiration is prolonged, (being frequently as long or longer than the inspiration,) generally more intense than the inspiration, rarely absent, the pitch *higher* than that of inspiration, and sometimes present without inspiration.

In *cavernous respiration*, the sound is likewise non-vasicular, with an interval between the inspiratory and expiratory sounds; the inspiration is blowing, low in pitch, and slowly evolved; the expiration being feeble, sometimes absent, but when present the pitch *lower* than that of inspiration.

There is a variety or modification of respiration frequently heard in the advanced stages of tuberculosis, and of this we desire to speak more particularly, at this time. Prof. Flint's attention was directed some years since to the coëxistence of bronchial and cavernous respirations in the same locality, in cases of phthisis more or less advanced. In his work above alluded to, this combination of the two kinds of respiration, he has styled, *broncho-cavernous;* in some cases the *bronchial* characters predominating, and in others, the *cavernous.* The reason for this will appear evident, when it is recollected that solidification to a greater or less extent exists around the cavity; and according to the size of the cavity, or the number of the cavities, and the amount of condensation surrounding them, does the respiration present more of the cavernous or more of the bronchial qualities. If there be one or two excavations of considerable size, and the surrounding lung be solidified to a small extent, then the characters of the former will predominate. If, on the other hand, the excavations are smaller, (although more numerous, perchance,) and the intervening tissue be solidified, the bronchial characters predominate.

The distinctive features of this variety of respiration may be stated as follows: the sound tubular or blowing, and an interval between the inspiration and expiration; the inspiration the same as that of the bronchial or of the cavernous, according to the predominance of the one or the other types of respiration, generally the qualities of the former more marked; the expiratory sound, however, possessing the most distinctive features. The bronchial respiration being more rapidly evolved than the cavernous, the first portion of the expiratory sound is generully bronchial; the cavernous respiration being rendered inappreciable by the intensity and rapidity of production of the former respiration. The *latter* portion of the expiratory sound is cavernous: the bronchial qualities having disappeared or been rendered inappreciable by the cavernous sound. This *change in pitch in the course of the expiratory sound,* is the distinctive feature of this modification of respiration. And this very peculiarity renders the *broncho-cavernous* respiration sufficiently easy for any practised ear to discover. From the physical conditions which give rise to it, it is met with very frequently; and when discovered, denotes not merely the fact of tuberculous solidification, but the actual existence of excavations. It is rendered still more valuable by the infrequent occurrence of a pure cavernous respiration. Having the present physical sign, we need not direct attention to the chest, in search of a purely cavernous respiration. In regard to its importance, the *broncho-cavernous* respiration is to the diagnosis of the advanced stages of tuberculosis, what the *broncho-vesicular* is to that of the earlier stages.

For the sake of convenience of reference, and in order to render the differ- between the several types of respiration and their combinations more striking, we have arranged them in a tabular form. Reference may be made to the appendix, for cases illustrative of this variety or combination of respiration. (Vide cases iv, v, vii and viii.)

*Tabular view of the Types of Respiration, and of their Modification.*

| | *Respiration.* | |
|---|---|---|
| *Vesicular.* | *Bronchial.* | *Cavernous.* |
| Normal. | Whenever there is solidification. | Rarely heard pure or uncombined. |
| *Sound:* Vesicular. | *Sound:* Tubular. | *Sound:* Blowing. |
| *Interval:* Does not exist between inspiration and expiration. | *Interval:* Exists between inspiration and expiration. | *Interval:* Exists between inspiration and expiration. |
| *Inspiration:* Vesicular in quality; low in pitch; longer than expiration; slowly evolved. | *Inspiration:* Tubular in quality; high in pitch; shortened in duration; rapidly evolved. | *Inspiration:* Blowing or non-vesicular in quality; low in pitch; and slowly evolved. |

| | | |
|---|---|---|
| *Expiration:* Lower in pitch than inspiration; shorter in duration, and less intense than inspiration; often absent. | *Expiration:* Tubular in quality; higher in pitch than inspiration; as long or longer than inspiration; generally more intense than inspiration; rarely absent; sometimes present without inspiration. | *Expiration:* Blowing in quality; lower in pitch than inspiration; shorter, and less intense than inspiration; sometimes absent. |

### *Modifications or Combinations.*

| *Broncho-Vesicular.* | *Broncho-Cavernous.* |
|---|---|
| Frequent; heard in the early stages; distinctive of more or less solidification due almost invariably to tubercle. | Frequent; heard in later stages; distinctive of cavity or cavities, surrounded by more or less solidification. |
| *Sound:* Mixed, tubular and vesicular. | *Sound:* Mixed, tubular and cavernous. |
| *Interval:* Exists between inspiration and expiration. | *Interval:* Exists between inspiration and expiration. |
| *Inspiration:* Composed of tubular and vesicular qualities; pitched raised; frequently shortened. | *Inspiration:* Composed of bronchial and cavernous qualities; usually the former predominating. |
| *Expiration:* Pitch somewhat higher than that of inspiration; prolonged and generally more intense than expiration; usually present, and sometimes present without inspiration. | *Expiration:* Pitch at first high, then suddenly becoming low; prolonged and non-vesicular; always present, in order to constitute this combination. |

*Effect of Hæmoptysis on the Progress of the Disease.*

One of the most frequent symptoms attendant upon pulmonary tuberculosis, is hæmoptysis.* It may occur at an early or at an advanced period of the disease, and even before the deposit of any tuberculous matter has taken place. It is a symptom which, when it occurs in an individual who had supposed himself unaffected with disease, attracts attention, and almost always leads him to ascertain the condition of the lungs.

The subject of hæmoptysis in its relation to tuberculosis, has been carefully investigated by Dr. Walshe. The conclusions he arrives at, are important. Only those which have an immediate bearing on the views we are desirous of developing, will be mentioned in this connection. For more extended remarks, reference may be made to Dr. Walshe's article, in the *British and Foreign Med.-Chirurg. Review,* for Jan., 1849.

Does hæmoptysis exert a favorable or an unfavorable effect on the progress and duration of tuberculosis, when it occurs in the early stages of the disease, or at any period when it may not be attributed to the rupture or ulceration of a blood-vessel of considerable size? When it occurs before the existence of any physical sign denoting a tuberculous deposit, that is, very early in the disease, does it exert any influence on the deposition of tuberculous matter? Whenever it takes place, but more particularly in the early periods of the disease, does it not prevent the deposit of tubercle which otherwise would have been formed?

These are important questions having a direct bearing on our therapeutical views; and they can only be answered by the results of clinical observation. Dr. Walshe found, that hæmoptysis occurred in 81 per cent. of the cases he examined; that it was more frequent in the second and third stages than previously; that it increased in frequency with advancing years; that this greater frequency in persons of more advanced years, did not depend altogether on greater duration of the disease; that the most common periods for its occurrence, were at the very outset or after the expiration of the first month; that season did not appear to exercise any marked influence on its

* It occurred in 91 of 100 cases analyzed by Prof. F. In 53 of these, it had taken place before the chest was examined; about 58 per cent. In 22 instances of small tubereulous deposit, it had occurred in 13; or 59 per cent. In 11 cases in which the existence of cavities was ascortained, hæmoptysis had been observed in 6, or about 54 per cent, And of 58 cases of abundant deposit, it had occurred in 34, or over 58 per cent.

first occurrence; that it never appeared as the *bonâ fide* first symptom in these cases, it being so only in the sense that it was the first occurrence that led them to watch their health. This last conclusion, he remarks, "leads one to reject the notion, that phthisis is really caused by hæmoptysis where it *appears* to be the first symptom." He adds, that "hæmoptysis is frequent in proportion to the duration of the primary disease." Hence, "we are forced to conclude, that *frequently-recurring hæmoptysis does not reduce the mean duration of life, after seizure with tuberculous symptoms, in any given mass of cases!*" Hæmoptysis, according to the same authority, may act as a local therapeutical agent; and that it is a mere coincidence when it occurs, and the disease *rapidly* proves fatal. These certainly are not fabled conclusions, and, if correct, having an important bearing on the treatment of phthisis.

For some time past, Prof. F. has expressed the belief, as based on his observations, that hæmoptysis was not an event to be dreaded; that it did not actually abridge the life of the individual; that, after its occurrence, the symptoms and physical signs did not indicate at the least, a worse condition of the patient; that in cases of frequently-recurring hæmoptysis, the patients live fully as long as, and even longer than, where this was not the case.

Still more recently, Dr. F., (from a more extended investigation of this subject,) has advocated the view, that hæmoptysis is to be regarded in a favorable light. We speak of it as occurring in the earliest periods of the disease, and when not excessive in quantity. It actually retards the progress of the disease. Why? Because we have every reason to warrant the opinion that the loss of a certain amount of blood is substituted for a deposit of tuberculous matter which would otherwise have taken place. The local hyperæmia of the lung has been relieved by the escape of the blood; if this congestion, which is supposed to exist generally, prior to the deposit of tubercle, had not been relieved in this way, then the deposition of a greater or less amount of tuberculous matter might have been anticipated. A few striking instances tending to illustrate these conclusions, will be found in the appendix. (Vide cases i, ii, and iii.)

*Period of Occurrence, and Duration.* In regard to the period at which pulmonary tuberculosis is most likely to occur, observers do not differ. It is rare to meet with this disease developed late in life. Two cases of this kind are related in the appendix, the patients being quite advanced in years. (Vide cases iii and iv.)

The duration of the disease is stated to be, according to M. Louis, in 307

cases, as follows: At the end of nine months, 147 alive, or about 48 per cent; at the end of two years, only 43 alive, or about 14 per cent.

Of the cases in the ward, which are now in the more advanced stages of the disease, the duration has been as follows: Four cases which were in the ward when the winter service commenced, are now in no immediate danger of a fatal termination; in one, the disease has existed seven years; in another, over five years; in a third, over three years; and in a fourth, over one year. Thus, we notice that the duration of the disease, under the present method of treatment, is much longer.

*Treatment.* The results of the treatment, or more appropriately of the management of the cases of tuberculosis in the ward, during the past winter, have been highly satisfactory. With the exception of a single instance, no patient has succumbed to the disease. This exception occurred in a person advanced in years, and who was admitted in a state of extreme prostration; he died about eight and forty hours after entering the hospital. Still farther, not a patient who has been under management more than a few weeks, has deteriorated in condition. Every case of this disease that has remained in the ward since the commencement of the winter service, (October 1st, 1857,) has either exhibited more or less improvement, or remained *in statu quo.* Although the number of cases is somewhat smaller than desirable, yet they have shown all the different stages of the disease. The foregoing results have not only attended cases of incipient tuberculosis, but likewise cases in which great portions of the lung were solidified or in which excavations existed.

Dr. Walshe states, that the number of patients leaving the hospital *improved* or *unadvanced*, was more than double that leaving it in a *worse state* or *dying within its walls.* "In above four per cent. of the cases, complete restoration to health, not only as regards apparent disturbance of the functions generally, but as regards local evidence of active pulmonary disease, was effected." "All patients whose condition grew worse, while they were in the hospital, had reached the stage of excavation on admission." "Improvement is more probable than the reverse, even where excavation exists on admission."

In what did the measures of management of the patients, under care this winter, consist? In the first place, *very little medication*, just sufficient to palliate or relieve unpleasant symptoms as they appeared; of which treatment, *anodynes* formed a considerable part. In the next place, *sustaining measures*, comprising a *generous diet* and the moderate use of *alcoholic li-*

*quors.* Very little resort has been made to *cod liver oil*, for two reasons: those who did make use of it, after a short time became tired of it, or complained of the nausea which it produced; and those who did not take it, exhibited a condition so satisfactory, as to render the employment of it quite unnecessary. No matter what may be said in its favor, cod liver oil is an unpleasant article to take, and when tolerated, the patient sooner or later longs for a change of medicine. It is still undecided whether this oil possesses any intrinsic value, or whether any other kind of oil, perseveringly employed, would not be attended with as much benefit as has been attributed to the use of the cod liver oil. Besides, there are other agents which we are able to employ, with as great, if not greater benefit. Of these, alcoholic liquors are the most important. *Whisky* has been the form generally made use of in the ward. It has been given in doses sufficiently large to exert some appreciable effect on the system; but not so large as to occasion any of its intoxicating effects, nor even to produce exhilaration of mind. In most of the cases under treatment this winter, this article has formed one of the chief measures of treatment. *Tonics* have sometimes been given, and have been continued for a length of time. They have been quite useful. The articles generally employed have been Huxham's tincture of bark, and quinia and iron.

The *diet* has consisted of meat, eggs, animal essences and broth, with milk, and farinaceous articles, and some vegetables. As small an amount of food not highly nutritious, has been allowed as has been practicable.

The symptoms most frequently calling for relief, have been cough, pain and diarrhœa. Anodynes have of course been employed, such as morphia. The cough syrup of the ward, (a solution of morphia in syrup of tolu,) has acted very well in the relief of cough and of pain.

By persevering in the medicines employed, and by strict adherence to the sustaining course of management, the condition of the patients has always improved, or at least has remained the same. For the most marked results attributable to this plan of management, see appendix, (cases vi, vii and viii.)

### *Acute Phthisis.*

A case of acute phthisis has come under observation this winter. This variety of tuberculosis is sufficiently infrequent to admit of our reporting the case at some length.

It will be seen, by a review of the symptoms, that the diagnosis of the case was not easy. It might readily have been mistaken for typhoid fever or for acute bronchitis. From either of these diseases it was distinguished by the absence of some of their diagnostic traits. The physical signs denoted merely bronchitis; and the true nature of the disease could only be determined by a careful consideration of the most prominent symptoms.

This form of phthisis depends on the presence of gray, semi-transparent granulations diffused more or less abundantly over the lobe of both lungs. This variation from the law of the disease as it usually occurs, renders the information derived from physical examination of the chest, comparatively unimportant. But, the rapidity of the pulse, the prostration of the patient, and the sudden and repeated attacks of difficulty of breathing, with the absence of much cough, great pain, or any amount of expectoration, are symptoms strongly indicative of acute phthisis, in any instance like the following.

Nov. 19th, 1857.* Case of J. W., German, aged 26 years, farmer; admitted yesterday afternoon. Has been five years in this country; enjoyed good health up to about six years ago, when he seems to have had an attack of acute rheumatism, which confined him to bed, some six or seven weeks. After recovering from this sickness, he continued well up to April last. He was seized with a cough, accompanied by considerable expectoration, but he did not suffer from pain in the chest. He continued to work about one month after he noticed the cough, but was obliged to discontinue his occupation on account of weakness.

About four months since, he appears to have had an attack of sub-acute rheumatism, which has continued up to the present time. This did not oblige him to take the bed, only occasionally. He has had cough constantly for about one month. He states that his parents and the members of their family, are well. None of them have ever been affected, to his knowledge, with any disease commonly esteemed hereditary.

His condition, on admission, was as follows:

Slight capillary congestion of face, diffused.

Aspect not morbid; mind clear.

Cannot sleep at night, on account of febrile movement; has no headache.

Had an attack of epistaxis, one week since; and has had it previously, after the act of coughing, when severe.

Tongue moist, not coated; occasional thirst.

Appetite good; no nausea nor vomiting at present; bowels regular.

---

* This case has not been included in the consideration of the foregoing subjects as it belongs to a peculiar and unusual form of tuberculosis. N.

Pulse 100, compressible, full, regular.

Cough frequent during the night; expectoration moderate in amount, and consisting almost entirely of clear mucus; has no pain in the chest at any time except after severe paroxysms of coughing.

Skin warm and moist; perspires whenever he falls asleep.

Has never had an attack of hæmoptysis.

Occasionally has suffered from sharp pain in the left side of the chest.

Is able to be up during the day; but feels very weak.

Urine is abundant, with some lateritious deposit.

On examination of the joints, both knees, both ankles, and both wrists were found to be swollen; there was no redness present; pain was only elicited by firm pressure and on motion. During the night, however he experiences pain in the muscles of both the upper and lower extremities.

November 30th. Since last record patient has continued much the same. An intermittent bellows murmur has been discovered, referrible to the aortic orifice; but now, it has disappeared.

December 18th. Patient has so far improved as to be able to sit up and to walk about, merely complaining of weakness. The treatment has been of late, quinia and opiates at night, with good diet.

January 1st, 1858. Patient suddenly attacked during the night, with sharp and stitch-like pains in both sides of the chest. There was much difficulty of respiration. The cough was frequent, and the expectoration was copious, but entirely composed of *clear mucus.* These symptoms continued at the time of the morning visit. Then, on examination of the chest, *no disparity* on percussion existed, and the resonance was not affected; *sibilant and sonorous râles* were heard universally over both sides of the chest.

These symptoms abated in a few days. But, on the night of January 8, the patient was attacked suddenly and presented the same symptoms as on the 1st instant. The expectoration consisted of nothing but perfectly *transparent mucus.* These symptoms became less marked in a short time. He kept the bed, however. His strength gradually failed. He began to emaciate rapidly. His features became pinched.

His condition on the 22d of January, was as follows:

Sleeps comfortably at night: complains of the febrile movement, however; has slight headache; feels very weak; emaciation considerable.

Aspect decidedly morbid.

Has considerable thirst; his appetite is good.

Has had, and still has *diarrhœa;* there is no pain in the abdomen.

Skin warm and mellow.

There is some loss of voice; has complained of pain referred to lower part of trachea.

Can swallow only a small amount at a time.

Complains of slight pain in the chest.

Pulse 120, quick, full.

Respirations 24, somewhat hurried; the superior and inferior costal types of respiration were marked.

Cough not severe; expectoration moderate, consisting almost entirely of clear mucus, occasionally a little blood, and an opaque sputum.

During the period of the sudden attacks of pain with hurried breathing, he exhibited some *delirium:* not only at night, but during the day, he occasionally muttered and talked incoherently.

Lately, however, his mind has been clear, and he has shown a singularly contented and tranquil disposition, making no complaints and being apparently resigned to his situation.

He continued to present the same category of symptoms: emaciation became extreme; he grew weaker and weaker; the pulse continued frequent; the expectoration diminished in amount, but its characters continued the same; and whenever the chest was examined, the dry bronchial râles were found everywhere present associated with no disparity on percussion, and no marked diminution of resonance. These were all the signs elicited, at any time, by repeated and careful physical explorations.

On the evening of the 8th of February, about five weeks after the first attack of pain, &c., he quietly died.

It remained only for a post mortem examination to confirm the correctness of the diagnosis. This, however, was not practicable. The case is interesting, on account of exhibiting in its course, so many resemblancos to acute bronchitis and to typhoid fever.

### APPENDIX.

CASE I. *Pulmonary Tuberculosis, accompanied by freuuent attacks of Sub-acute Rheumatism; Repeated attacks of Hæmoptysis; Disease, singularly non-progressive; Effect of the Iodide of Potassium.*

April 12th, 1855, T. C., aged 30, Irish, tailor.

*Previous History.* He states that when he was about 16 years of age, he had an attack of hæmorrhage; from his account, it was impossible to ascertain whether the blood came from the lungs or from the stomach; he attributed the occurrence to over-exertion. After this even the enjoyed good health up to a little less than two years ago, with the exception of frequent attacks of sub-acute rheumatism, which he has been subject to since he was 16 years of age. Summer before last he had a cough, which was slight, and to which he paid but little notice. He thinks that he lost flesh during that summer. In the autumn following he had an attack of *hæmoptysis*, considerable in amount; and the hæmorrhage continued off and on for about three weeks. He had a slight recurrence of hæmoptysis last summer; with this exception, it has not occurred since a year and a half ago. After the attack of hæmoptysis, autumn before last, he went to Tennessee, and remained there till last May, nearly a year since. During the winter he was South, had more or less cough and expectoration. Has been in this city since last May. He has had more or less cough and expectoration during last summer, and was able to work at his trade but a portion of the time. During last winter, he was unable to work. The cough and expectoration continued. He has been troubled much of the time with rheumatism affecting the joints, sub-acute in character. He is now laboring under an attack of this kind, affecting the joints of the phalanges and metacarpus. The rheumatism has confined him to bed. Aside from this, he is able to be up and about. He has lost considerable flesh, but he does not know the amount.

*Present Condition*, He does not present an appearance of emaciation, nor is his aspect notably morbid. Appetite capricious; no diarrhœa, except for a short time last summer; skin cool; has had chills occasionally; Pulse 80; feels the want of breath on active exercise; the respirations are not accelerated; there is considerable expectoration, which is muco-puruloid; he has but little cough.

*Physical Signs*. Evident depression below the right clavicle; dullness marked at the summit of the right chest, and also over the right scapula; no disparity, on percussion, below scapula. In right infra clavicular region, the inspiratory sound is much less developed and shorter than on the left side; it is too feeble to determine satisfactorily the pitch and the quality. On the left side, it is well developed, prolonged, and vesicular in quality. A prolonged, relatively high-pitched expiration exists on the right side; but no expiratory sound is heard on the left side. Local resonance is greater at the right than the left summit.

He has been placed on the use of cod liver oil.

April 13, 1856. This patient has been in the hospital for the past four months, and has suffered more or less from rheumatism during that time.

On physical exploration of the chest, the signs denoting solidification at the right lung, were more decided. Besides, there were present the moist and dry bronchial râles limited to the summit of the same lung. There were no signt present indicative of an excavation.

May 14th. Sat up.

He has lately been taking the iodide of potassium; whenever this has been administered, the rheumatic affectiou has been relieved, but there has appeared on the face and neck, a papular eruption, which soon disappears when the remedy is discontinued, and when continued becomes vesicular and pustular.

On the 25th of May he left the hospital.

November 6th, 1856, he was reädmitted with an attack of sub-acute rheumatism. He has had frequent attacks of rheumatism since he left the hospital. Has been at work part of the time. The cough has continued, and the expectoration is now greater than at any time heretofore, and consists of distinct, opaque sputa. There has occurred *slight hæmoptysis*, twice during the summer. His aspect is morbid; has lost some flesh since May last, but is not emaciated. The pulse is 64; respirations 16.

On examination of the chest on the 14th of November, the physical signs denoted no great extension of the disease on the right side, and no excavation existing; but they showed that the summit of the left lung, heretofore unaffected appreciably, was now more or less solidified by tuberculous deposit.

January 7th, 1857. This patient continued to take the iodide of potassium from the 28th of November until the 14th of December, in five grain doses, three times daily. On the latter day the dose was increased to ten grains; this was continued until the 18th of December. On the 18th and 19th, having complained of nausea, and having vomited, the medicine was discontinued. Its use had been attended with the papular eruption so frequently observed previously. On the 20th the sub-nitrate of bismuth was administered. On the 21st, the patient presented all the appearance of mercurial *salivation*, except that the fetor of the breath was not so marked as in ptyalism from a mercurial. The bismuth was examined by Prof. Hadley, and found to be free from any impurity. The mouth soon became sore; the gums were tender and separated from the teeth; the teeth became loose; small ulcers formed upon the mucous membrane; and the quantity of saliva poured out was great. The rheumatic affection rapidly disappeared; and the cough, expectoration, and pain in the chest, were notably diminished. From this ptyalism he gradually recovered. It is a fact worthy of note, that the salivary glands did not appear to be affected during this salivation.

April 22d, we find the patient relieved of the ptyalism, up and doing light work about the ward. He now complains of pain in the chest, which, on examination, proves to be an attack of intersostal neuralgia.

He was soon relieved of this pain, and during the summer continued in the hospital. He suffered occasionally from attacks of sub-acute rheumatism. The cough and expectoration continued.

November 14th. This patient had an attack of hæmoptysis, a considerable amount of blood was lost, about half a pint.

December 30th. For a few days past has been complaining of rheumatism in the joints. No iodide of potassium has been given for a month. To-day, the papular eruption has made its appearance, although not as abundantly as usual. The rhematic attack is abating.

(The above fact is interesting, on account of the appearance of an eruption supposed to be produced by the iodide of potassium, when over four weeks had elapsed since any of that article had been used. Here is another instance of the caution to be observed in drawing conclusions respecting the effects of therapeutical agents in the treatment of disease.)

January 24th, 1858. His general health is as good as when seen in October last. He is not emaciated. He is free from rheumatism. He has but little cough, and occasional expectoration. He complains of no pain in the chest. His pulse and his respirations are not accelerated. His aspect is improved. he has no diarrhœa.

Case II. *Pulmonary Tuberculosis; Very frequent attacks of Hæmoptysis; Fistula in ano. Slow Progress of the Pulmonary Disease.*

December 9th, 1857. M. S. Q., aged 33, seaman, colored; has been in the hospital about two weeks.

*Previous History.* States, that up to five years since, considered himself perfectly well. The first symptoms that attracted his attention, were coughing and expectoration, but accompanied by no hæmoptysis. This was in March, 1853. He weighed then 108 pounds. The character of the expectoration was distinct, opaque, yellow sputa, with some clear mucus. Frequently suffered from stitch-like pains in the summit of both lungs.

About four or five weeks after cough commenced, had an attack of hæ-

moptysis. The blood was expectorated without any previous pain, and did not exceed a tablespoonful in amount. These attacks of hæmoptysis have continued more or less ever since: on the average, he thinks, at least once every week. Soon, he commenced to perspire at night, and to loose flesh. Did not notice any loss in strength, and did not take to the bed until the appearance of the fistula. The cough and expectoration continued. Did not have attacks of diarrhœa. Felt unwell a day or two prior to the formation of the fistula. The fistula commenced as an ordinary boil; this discharged a considerable quantity of matter and there has been more or less discharge from the fistula ever since. The fistula made its appearance about one year since. Another fistula soon formed on the same side of the anus, but nearer to the nates. Both of these are incomplete fistulas. He has continued to work much of the time since the fistula appeared. After the fistula had been formed, he noticed the cough to become less severe, and the expectoration to diminish in quantity; the attacks of hæmoptysis were also less frequent.

*Present Condition.* Aspect not morbid; not much emaciated; weighs now 170 pounds. Has no night sweats. Sleeps well. Feels weak; is up and about all of the day. Appetite fair; bowels regular. Does not complain of any pain in the chest. Has frequent cough: the expectoration is slight and consists of yellow sputa mixed with a little blood; expectorates a small quantity of blood almost every day. Pulse well-developed, slow, 72. Respirations tranquil, and number 16. Skin warm. The fistulas discharge considerably at present.

*Physical Exploration.* On the left side, in the infra-clavicular region and upper scapula, vesiculo-tympanitic resonance on percussion. Greater vocal resonance in same regions, on the left, than on the right side. A sibilant râle confined to same regions on the left side. Broncho-vesicular or rude respiration, on left side, in these regions; on right side, same regions, vesicular respiration. Greater expansion of infra-clavicular region on right side. In lower portions of chest, in front and behind, no disparity on inspection or by percussion; vesicular respiration heard on both sides, below.

CASE III. *Pulmonary Tuberculosis: Developed in Middle Period of Life; Several Attacks of Hæmoptysis; Slow Progress of the Disease.*

February 3d, 1858. J. C., Irish, aged 50, laborer. States, that he considered himself well up to about six years ago: noticed then, that he had a dry, hacking cough, accompanied by no expectoration, neither any pain in the chest. The cough soon became more severe, and a slight expectoration appeared. Observed that he became short of breath on active exercise. He began to lose flesh and strength. Suffered from night-sweats. About five years since, was attacked with intermittent fever, from which he has never been entirely free, since; has a paroxysm every few weeks, or oftener. Soon after he was attacked with intermittent fever, noticed a swelling in the right lumbar region which was opened and discharged a considerable quantity of pus.

*Present Condition.* Aspect sallow, unhealthy. Has lost about thirty pounds in weight, Feels weak, and cannot do a hard day's work, but is able to be up and about all day. Appetite poor; bowels regular; has had no diarrhœa. Pulse 84, quick and small. Respirations somewhat labored, and number 24. Has no pain in the chest now. The cough is frequent; expectoration copious, and consisting of yellow, opaque sputa. Says, that within the past six years he has had three attacks of hæmoptysis: the last occurred about two weeks since; expectorated a large amount of blood. The abscess in the right lumbar region has not discharged since November last. He complains most, at present, of shortness of breath on moderate exercise.

*Physical Signs.* The physical signs showed tuberculous solidification, somewhat extensive, at the summit of the right lung; bronchial respiration existed in the infra-clavicular region of this side; the other signs were very similar to those recorded in Case II, as found at the summit of the left lung; there were no physical signs of excavation.

CASE IV. *Pulmonary Tuberculosis: Developed in Advanced Life; Stage of Excavation; Broncho-cavernous Respiration.*

February 7th, 1858, J. S., Irish, aged 78. Admitted on the 2d instant, very much prostrated. His feeble condition prevented our obtaining a full account of his previous history.

He stated that he was attacked with cough and expectoration about eighteen months ago, which have continued ever since; he has suffered from sharp pain in the chest. Prior to eighteen months since, considered himself well. He was very much emaciated. The cough was frequent and severe. There was a copious expectoration of clear mucus, and of thick, yellow sputa. His pulse was quick and small, and rapid. The respirations were hurried.

On examining the chest, anteriorly, on the right side, in the infra-clavicular region, between the 2d and 3d ribs, a tympanitic sound on percussion, ut no *bruit de pot fêlé; broncho-cavernous* respiration well marked in same locality; elsewhere, in same region, bronchial respiration; on left side, same region, broncho-vesicular respiration. Greater expansive movement of *left side*, *most* marked in infra-clavicular region.

CASE V. *Pulmonary Tubercnlosis affecting one side much more than the other; Bulbous Enlargement of the Finger-ends; Broncho-cavernous Respiration; Stage of Excavation.*

October 2d, 1857. P. C., aged 16, laborer, has been in the hospital since the 23d of last May.

The *previous history* is as follows:

None of his immediate relatives have died of tuberculosis. Up to February last, enjoyed tolerable health: during the winter, however, he had a cold, and noticed that he was losing flesh. Had no attack of hæmoptysis up to period of admission. Kept at work, being much exposed to the cold. Took to the bed in February last, on account of pain in the right side of chest, accompanied by cough and expectoration. Since his admission has had one attack of hæmoptysis.

*Present Condition.* Considerable emaciation; profuse night-sweats. Cough frequent; expectoration copious and muco-puruloid; has not much pain in the chest. Skin warm; respirations 20; pulse 80. Appetite fair; no diarrhœa; bowels regular. Feels very weak, and suffers from shortness of breath. Fingers and toes clubbed at their ends with curved nails.

*Nov. 22d, Physical Examination of the Chest.* The left side presents notably greater expansion on forced breathing. The right side, in front, gives no vesicular resonance on percussion, whilst this quality of sound is sufficiently marked on the left side. The resonance, in front, at upper third, on the right side, is tympanitic. In the 2d intercostal space, on the right side, the respiration is *broncho-cavernous*, and on percussion, a well-marked *bruit de pot fété; no pectoriloquy,* but at right summit broncophony is marked, &c.

January 26th, 1858. This patient has been taking cough syrup and milk punch. He has been up, frequently, in the afternoon. He has a copious expectoration. His appearance is materially improved since last October. There are no night-sweats, now. His strength does not appear to be failing; and the *emaciation is not* marked.

Case VI. *Pulmonary Tuberculosis: Intense Bronchial Respiration; Remarkable effects of Treatment.*

November 3d, 1856, P. C., Irish, aged 51 years, laborer; admitted October 27th.

*Previous History.* Considered himself perfectly well up to March last. His father died with phthisis, and is brothers have been subject to it. Was suddenly attacked, after exposure to cold and wet, with a sharp pain in the left side of chest, accompanied by cough. This cough, he says, he has had for some years, but has paid no attention to it. The next day, he observed a slight expectoration. He took to the bed, and kept it most of the time, until he came to the hospital in May last. His cough grew more and more severe. The expectoration became much more copious, thick, yellow, and fœtid. The pain in the left side continued more or less severe. He was obliged to lie on the right side. Grew weaker, lost considerable flesh, and had profuse perspiration at night.

Remained in the hospital about two weeks; he was much improved in strength, and the pain was relieved; the cough and expectoration diminished. He was able to get out of bed, and walk about the ward. He left the hospital; and soon the cough and expectoration and pain returned. Was obliged to take to the bed; continued to lose strength and flesh. And entered the hospital in the latter part of October.

When first seen, was extremely prostrated. Face was pallid; there was profuse perspiration; he could lie only on the left side. The pulse small, and 100 per minute. Respirations 32. The cough frequent and severe: the expectoration very abundant and extremely offensive, consisting of thick yellow sputa; he had expectorated about a pint in the twenty-four hours. He complained of severe, stich-like pain in the right side, increased by forced respiration.

He was placed on the use of brandy: and has improved materially.

*Nov. 6th, Chest examined to-day.* Dullness, amounting nearly to flatness, over whole of left side, anteriorly, laterally, and posteriorly: clear, resicular resonance on right side. Intense bronchial respiration, every on the left side, in front, laterally, and behind. Bronchophony is intense every where on the same side; the whispering souffle also is intense, and near the ear, every where on left side. On the right side, the vesicular murmur is well evolved; with a sonorous râle occasionally, both at the summit and at the base. At the summit, in front, a prolonged and high pitched expirabion is heard, over middle of infraclavicular region. The right side expands somewhat more on forced breathing than the left.

Nov. 16th. This patient is improving: his aspect is better. The expectoration is copious, and of same appearance as heretofore, but has lost its fœtid odor.

He is taking half a pint of brandy in the twenty-four hours, with cough syrup: the diet is sustaining.

Nov. 22d. This patient sat up about an hour yesterday. The treatment now consists of

Cod liver oil f. ℥ ss, 3 times,
Brandy f. ℥ ss, every two hours, and
Cough syrup f. ʒ i., every two hours, and sustaining diet.

Jan. 7th, 1857. The patient has continued on the above treatment with the exception of morphia, grain $1\frac{1}{4}$, in place of the cough syrup: this medicine has gradually been increased, until he is taking now two grains at bed time. His aspect is much better. He enjoys refreshing sleep at night. The cough is less frequent: and the expectoration is diminished in quantity and not offensive; the characters continue the same. His appetite has been fair: he has had two attacks of diarrhœa lately.

February 1st. On examining the chest, this morning, the same physical signs were observed as at the last record. But in the upper scapular and upper part of the inter-scapular regions, the quality of respiration was *broncho-cavernous*. On the right side, over and below the scapular, the respiration is broncho-vesicular. On the left side, the sound on percussion, is tympanitic; but no distinct *bruit de pot fêlé*.

This patient presented a very much better condition on May 2d, (the termination of the winter service,) than when admitted. He terminated his life, on the night of the 10th of July, 1857, by his own hands, during tem porary aberration of mind.

Case VII. *Pulmonary Tuberculosis; Slow or Non-progressive; Broncho-Cavernous Respiration; Effect of Hygienic Measures and of Treatment.*

December 7th, 1856. B. D., Irish, aged 30, laborer.

*Previous History.* States that he has had repeated attack of intermittent fever within the past four years. He noticed cough first in January, 1855, and suffered from stitch-like pains at the summit of the left side of the chest. During the winter, he expectorated considerable; and sometimes had hæmoptysis. At no time was he confined to bed. Kept at work most all the time, occasionally losing a day or so. The cough continued until the following May. Is not aware that he lost any flesh. From May, 1854, to February, 1856, was free from cough; considered himself well; and worked steadily. Began to cough again in February, 1856. And in March and April, was occasionally obliged to keep the bed. He never had any medical attendance, nor did he take any medicine prior to entering the hospital. Last May he was bled by some one to whom he applied. Continued to work, more or less, during the summer. Has been accustomed to the use of whisky. He thinks that he has not lost any weight within the past two years.

*Present Symptoms.* Aspect not unhealthy; he is not emaciated. He states that he feels strong and able to work, but he suffers from shortness of breath, and from stitch-like pains at the summit of the left side. Appetite good: bowels are regular: occasionally, has had attacks of diarrhœa. The cough is frequent: the expectoration is moderate in amount. Respirations 20. Pulse 84.

Was placed on the use of cod liver oil.

December 13th. Cough and expectoration diminished. Feels able to return to work; and leaves the hospital to-day.

Prior to his departure, the following physical signs existed, on examination of the chest:

Slight lateral spinal curvature, to right side. More expansion of chest, on right side, above, and anteriorly; greater elevation of right scapula: more marked on forced breathing.

On percussion, in front, on left side, in supra and infra-clavicular regions, high pitched and tympanitic resonance. At left summit, in front, no respiratory murmur, except at acromial extremity of clavicle, where it is bronchovesicular. On right side, at summit, in front, respiration vesicular and well evolved.

Reëntered the hospital on the 14th of October. States that he has been at work ever since leaving the hospital. The cough and expectoration have continued ever since; and he has some hoarseness of voice. Feels weaker than when he left the hospital.

He has had night-sweats, and has lost considerable in weight during the summer. Has suffered more or less from the stitch-like pain in left side of chest. Now he is able to be up all day and out of doors; but feels too weak to work. Finds himself short of breath on exercise.

His appetite is good: but has had frequent attacks of diarrhœa during the past summer. Pulse 100. Respirations 20.

Complains, now, most of pain in the left side of the chest: cough, expectoration, and night sweats continue; he is somewhat emaciated. Otherwise, he is in much the same condition that he was last winter.

The treatment is to consist of cough syrup, cod liver oil, and morphia at night.

*Nov. 30th. Examination of Chest.* Marked depression at the left summit in front. Less expansive movement on left side, in front, in infra-clavicular region: and less elevation of left scapula, on forced breathing. Great dullness at upper and middle thirds, in front and behind, on left side: in front, this is of a tympanitic quality. On right side, vesicular resonance in front and behind. On same side, inspiratory sound vesicular, without any expiratory sound. On left side, in front, at the summit, *broncho-cavernous* respiration. Behind, on same side, respiratory murmur obscured by bronchial râles.

December 13th. Patient had an attack of hæmoptysis last night, inconsiderable in amount. It is now fourteen months since the last attack.

His general appearance is better than when he entered the institution. Is up the entire day, and able to be out of doors. His voice is not as husky as when he entered.

He is taking now:

Cod liver oil, milk punch, morphia, grain ¼ at night; and he is on the use of a sustaining diet.

Case VIII. *Pulmonary Tuberculosis; Broncho-Cavernous Respiration; Slowly Progressive; Effects of Treatment.*

March 1st, 1857. E. H., Irish, aged 55, laborer.

Previous to six years ago, considered himself perfectly well. During the winter of 1851 and '52, noticed a cough, accompanied by slight expectoration; about two months after this, was attacked with hæmoptysis; expectorated the first time less than a teaspoonful. Did not suffer from any pain in the chest. Lost a little in weight. In the following spring, felt much better; the cough and expectoration disappeared. Kept at work all the time During the winter of 1851 and '52 had, at the least, half a dozen attacks of hemorrhage from the lungs.

From the spring of 1852 to the winter of 1856, over four years, he seems to have been free from any pulmonary symptoms. But on the approach of that winter, the cough and expectoration returned. There has been no return of the hæmoptysis. He has had night-sweats. Has lost considerable in weight during past winter. Has suffered from pains in the chest, but none at the summit.

Now, his aspect is pallid: there is some emaciation; has most cough and expectoration in the morning; the latter is not copious, and consists of opaque, yellow sputa, with some transparent mucus. Perspires at night. The appetite is good: bowels costive. Respirations 16, and pulse 76.

*Physical Examination.* Right side, at summit, flattened; superior costal movement greater on the left side. On percussion, distinct relative dullness on right side, at the summit, in front, and over scapula, behind. On the right side, every where, sonorous and sibilant râles, more or less constantly present, but not intense. Occasionally, at left summit, a feeble sonorous râle with the expiration; local resonance greater on the right side.

The treatment consisted of:

Cod liver oil f. ℥ ss, 3 times,
Cough syrup; and regulated diet.

May 1st. Patient remains in about the same condition.

October 4th. Since last May, has not been confined to bed all the time; has been in bed, now, about three weeks. He has lost some in weight, and is much weaker. Has suffered from shortness of breath. Has not had much pain in the chest. During the past summer, has expectorated blood several times. He has had but little cough and expectoration. Has not perspired at night lately. Appetite continues good; the bowels are regular; and there has been no diarrhœa. Pulse 100. Respirations 24.

On examination of the chest, the physical signs showed no great extension of the disease on the right side; but on the left, the disease had proceeded to the formation of excavations, as proved by the marked tympanitic resonance on percussion, and more especially by the presence of the *broncho-cavernous* respiration on the left side, at summit.

He is placed on the use of:

Whisky, cough syrup, and occasionally cod liver oil.

January 26th, 1858. His general appearance has materially improved. His cough and expectoration have been slight during the winter. He has never complained of pain in the chest. He has had no attacks of hæmoptysis. His mind has been composed: and generally, he has been sleeping at the time of the daily visits. He has been up and dressed several times this winter.

